# Confidential enquiry is associated with reduction in malaria deaths in Mali

**DOI:** 10.1186/1475-2875-13-S1-P106

**Published:** 2014-09-22

**Authors:** Merlin Willcox, Drissa Diallo, Chiaka Diakité, Pierre Daou, Drissa Bamba, Eugene Dembélé, Anthony Harnden, David Mant

**Affiliations:** 1 University of Oxford, Oxford, UK; 2 Université des Sciences, Techniques et Technologies de Bamako, Bamako, Mali; 3 Institut National de Recherche en Santé Publique, Bamako, Mali

## Introduction

Malaria remains an important cause of childhood deaths in Africa, although effective preventive measures and treatments exist. Confidential enquiry has been used as a method for investigating childhood deaths in the UK and suggesting improvements to the health system. We aimed to adapt this process for sub-Saharan Africa, and to pilot whether it could be used to reduce mortality.

## Methods

A system for reporting under-five deaths was set up in two areas of Mali, starting in August 2011. An informant in each village calls a fieldworker, who comes to interview the family of the deceased, as well as any health workers involved. This information is reviewed by a multidisciplinary panel which diagnoses the most likely cause of death, identifies avoidable factors and makes recommendations for avoiding such deaths in future. Results were disseminated to communities. Case discussions were used for continuing professional education of health workers.

## Results

From August 2011 to August 2012, there were 81 and 161 under-five deaths reported in Massantola (Kolokani) and Finkolo (Sikasso) subdistricts respectively. 53 (65%) and 67 (42%) of these deaths respectively were attributed to malaria. The number of deaths was 2 - 4 fold higher than predicted by official statistics. In 89% of the malaria deaths, the family claimed that the child had been sleeping regularly under a mosquito net.

54% of patients had received home treatment, and 95% attempted to seek treatment outside the home, although this was delayed in about one third. Of those who sought care, the majority went to a public community health centre. Only 9% of these received adequate quality of care. Quality of care was inadequate at all other levels, including traditional healers, private health workers and hospitals. In the second year of the confidential enquiry, the number of deaths in each area has reduced by about 30%.

## Conclusion

Malaria is still the most frequent cause of under-5 deaths in Mali. Almost all of these deaths are avoidable. The single most important avoidable factor is quality of care at all levels. The confidential enquiry was associated with a 30% reduction in under-5 deaths.

